# Associations of the COVID-19 pandemic with social well-being indicators in Mexico

**DOI:** 10.1186/s12939-022-01658-9

**Published:** 2022-05-21

**Authors:** M. Vilar-Compte, M. Hernández-F, P. Gaitán-Rossi, V. Pérez, G. Teruel

**Affiliations:** 1 Department of Public Health, Montclair State University, 1 Normal Avenue, Montclair, NJ 07043 USA; 2 Research Center for Equitable Development EQUIDE, Universidad Iberoamericana, Prolongación Paseo de la Reforma 880, Lomas de Santa Fe, 01219 Mexico City, Mexico

**Keywords:** COVID-19, Mexico, Income loss, Employment, Food insecurity, Anxiety

## Abstract

**Background:**

Public health measures for COVID-19 containment have implied economic and social life disruptions, which have been particularly deleterious in low- and middle-income countries (LMIC) due to high rates of informal employment, overcrowding, and barriers to accessing health services, amongst others social determinants. Mexico, a LMIC, is a country with a high COVID-19 mortality in which there has been a very limited governmental response to help mitigate such COVID-related disruptions. This study analyzes the association of the first wave of the COVID-19 crisis in Mexico with four well-being indicators: income, employment, anxiety, and food security.

**Methods:**

It uses pooled cross-sectional data (*n* = 5453) of five monthly nationally representative surveys collected between April and August 2020. Probit models are estimated to assess the association of the pandemic with job loss and anxiety; a multinomial logistic regression is estimated for food security, and an ordinary least squares regression assesses the association between the pandemic and changes in household’s income.

**Results:**

Females were significantly associated with worse outcomes for the 4 well-being measures with an average reduction of 2.3% in household income compared to pre-COVID-19 levels, an increased probability (6.4 pp) of being in a household that had lost jobs, decreased probability of food security (6.9 pp), and an increased risk of anxiety symptoms (8.5 pp). In addition, those with lower SES and household with children also reported worse outcomes for employment, income and food security. The month variable was also statistically significant in these models suggesting that as more months of the pandemic elapsed the effects persisted.

**Conclusion:**

The currents study documents how the COVID-19 pandemic is associated with different well-being indicators in a LMIC. It suggests the urgent need to take actions to support vulnerable groups, particularly women, households with children and those in the lowest SES. If policy actions are not taken, the pandemic will increase social and gender disparities, and will jeopardize childhood development.

**Supplementary Information:**

The online version contains supplementary material available at 10.1186/s12939-022-01658-9.

## Background

Together with the fear of COVID-19 as a new disease, the public health measures to contain the pandemic have implied high degree of social uncertainty and have affected diverse well-being indicators [4]. The pandemic is having sustained effects on income and consumption. According to a global study [35] this will increase poverty levels. In some regions the adverse impacts could result in poverty levels similar to those reported 30 years ago, hence profoundly impacting the fulfillment of the Sustainable Development Goals (SDGs) and reversing decades of progress in poverty global reduction [35].

International agencies and academic communities alike have warned that food insecurity will most likely be a key consequence of the COVID-19 pandemic [11, 36, 37]. Studies are confirming such trends (see Table 1). In the US, it has been documented that the pandemic has disrupted food access and impacted food insecurity [28], especially among those who were already vulnerable pre-COVID-19 [10] or that have suffered impacts on different well-being indicators [33]. The effects of the pandemic have also been documented in other countries such as Brazil [25], and Mexico [14]. There is also evidence of a matching effect in food insecurity and mental health. For example, a study in Bangladesh, revealed that during lockdown measures there was a parallel increase in food insecurity and depression [17]. Another study in the US revealed that during the initial stages of the pandemic food insecure households were 2.09 and 1.88 times more likely to report anxiety and depression, respectively [22].

**Table 1:** Characteristics of individuals in the analytic sample, ENCOVID-19

	April	May	June	July	August	All
Sample size (n)	762	598	1435	1338	1320	5453
Gender, % (n)						
Male	49 (336)	49 (268)	49 (656)	47 (612)	48 (639)	48 (2511)
Female	51 (426)	51 (330)	51 (779)	53 (726)	52 (681)	52 (2942)
Anxiety, % (n)						
No symptoms	68 (514)	72 (425)	67 (963)	67 (921)	67 (899)	68 (3722)
With symptoms	32 (248)	28 (173)	33 (472)	33 (417)	33 (421)	32 (1731)
Socioeconomic status, % (n)						
E (low SES)	8 (67)	13 (45)	13 (91)	9 (65)	10 (85)	10 (353)
D (low-medium SES)	41 (318)	48 (258)	45 (581)	53 (556)	51 (549)	48 (2262)
C (medium SES)	43 (316)	34 (260)	37 (665)	34 (599)	35 (597)	36 (2437)
A/B (high SES)	7 (61)	5 (35)	5 (98)	4 (118)	3 (89)	5 (401)
Food insecurity, % (n)						
Food secure	40 (310)	36 (233)	30 (465)	25 (392)	25 (416)	30 (1816)
Mild food insecure	33 (255)	4 (244)	4 (576)	46 (612)	44 (572)	41 (2259)
Moderate food insecure	17 (125)	12 (62)	19 (256)	17 (205)	19 (211)	18 (859)
Severe food insecure	10 (72)	11 (59)	11 (138)	12 (129)	12 (121)	11 (519)
Job lost by a household member, % (n)						
No	63 (482)	75 (434)	73 (1012)	71 (968)	68 (925)	70 (3821)
Yes	37 (280)	25 (164)	27 (423)	29 (370)	32 (395)	30 (1632)
Household size, mean (se)	4.6 (0.1)	3.5 (0.1)	3.6 (0.1)	4.04 (0.1)	3.98 (0.1)	3.94 (0.0)
Age, mean (se)	40 (0.5)	41.6 (0.8)	41.3 (0.5)	40.07 (0.5)	40.09 (0.5)	40.57 (0.2)
Percentage change in household income, mean (se)	−26.3 (1.3)	−28.5 (1.5)	− 28.4 (1)	− 29.09 (1)	−27.91 (1)	− 28.17 (0.5)

Other studies have centered in solely documenting the toll of the pandemic on mental health. For example, a cross-sectional study in Italy found that after the first wave of the pandemic, 5.1% of the population showed post-traumatic stress disorder symptoms and 48.2% lower psychological well-being linked to the COVID-19 [12]. Similarly, a cohort study in the United Kingdom comparing tendencies pre and post COVID-19 first wave, showed that mental health deteriorated [32]. This coincides with a panel of experts who suggested that due to the pandemic and its associated economic downturn there is a risk that the prevalence of people with anxiety, depression and engaging in harmful behaviors will rise, as this has been a trend in prior epidemics [19]. Furthermore, it is important to consider that the pandemic can have an unequal effect on societies, as the crisis can increase disproportionately unemployment, financial insecurity and poverty among those who were already vulnerable, hence, placing them at higher risk of mental health conditions.

In terms of the inequities that are unfolding with the pandemic, it is of key relevance to underline that following the recommended public health measures in low- and middle-income countries is challenging, due to factors such as high rates of informal employment, suboptimal housing conditions, and low quality basic services such as running water, drainage and waste collection amongst others [8]. When such social determinants are in place, complying with social distancing and quarantine is more difficult. For example, for many who are informally employed there has been no option rather than showing up to work, as their daily wages are used for subsistence. Not recognizing these dynamics jeopardizes the survival of large segments of vulnerable populations [8].

Mexico is an upper-middle income country facing one of the highest COVID-19 mortality. According to data from *Worldometer* on March 2022, more than 320,400 deaths had been reported, only below the US, Brazil, India, and Russia. Excess mortality data analyses suggest that mortality has been underestimated, most likely, due to the low levels of testing in the country [26]. Households in Mexico, as in other low- and middle-income countries, share social determinants that can magnify the impact of the crisis in well-being such as a fragmented health system with lack of adequate public investment, large income inequalities, high levels of poverty, a large informal economy, and high levels of unplanned urbanization with low quality basic services.

While other Latin American countries expanded social assistance to offset the impacts of the pandemic [23], in Mexico there has been a very slow and limited expansion of social assistance [24], exposing the population to undesirable side effects of the pandemic and the public health measures implemented. For example, a report assessing the adoption of social policies during the pandemic in the Latin American and Caribbean region, showed that Mexico was one of the only countries that did not expanded the payments or coverage of pre-existing cash-transfer programs [7]. Moreover, prior studies documented that during the pandemic the Mexican Federal Government adopted a fiscal austerity line which affected salaries, infrastructure, programs, and public agencies that could have helped handling the pandemic and its deleterious economic and social impacts [34].

The aim of this study is to describe the association of the first wave of COVID-19 crisis with four well-being indicators in Mexico – employment, income, anxiety and food security – and assess the role of pre-COVID vulnerabilities (i.e. socioeconomic level) on such outcomes. The selection of these well-being indicators is an effort to capture some of the far-reaching consequences of the COVID-19 pandemic on the economic, social, and health support systems that have been disrupted during the pandemic [34].

## Methods

### Design and data

This study is based on a pooled cross-sectional analysis of five monthly waves of the ENCOVID-19, a nationally representative telephone survey of Mexicans 18 years and older who have a mobile phone. The ENCOVID-19 was first fielded in April 2020 and it will be collected at least until December 2021(Pérez Hernández, [30]. Encuesta Nacional sobre los Efectos del COVID-19 en el Bienestar de los Hogares Mexicanos (ENCOVID-19-JULIO) (Version 1)). It collects data on employment, income, food security and mental health to document the impacts of the pandemic and to inform key stakeholders. The survey follows a one-stage stratified probabilistic sampling of mobile telephone numbers which are randomly selected from the publicly available National Dialing Plan [20]. As of 3 April 2020, the coverage of mobile phones in Mexico was 96% [1]. Sampling weights correct for minor deviations from the Mexican population’s demographic structure. Weights are estimated using the 2015 mid-census survey data from National Bureau of Statistics (INEGI for its acronym in Spanish) and adjust the sample by state, gender, age, and socioeconomic status. Further details of ENCOVID-19 and the composition of the sample are available elsewhere (Pérez Hernández, [30]. Encuesta Nacional sobre los Efectos del COVID-19 en el Bienestar de los Hogares Mexicanos (ENCOVID-19-JULIO) (Version 1)).

Data is collected by trained interviewers using Computer Assisted Telephone Interviewing software (CATI). An assistant supervised the quality of interviews using the CATI software. On average, the interview takes 18 min. The data collection periods and analytical sample used in the current study are: April 6–14 (*n* = 762); May 20–25 (*n* = 598); June 5–17 (*n* = 1435); July 8–17 (*n* = 1338), and August 19–2 Sept (*n* = 1320).

### Measures

The study assessed the associations of the pandemic on four dependent variables: employment, income loss, anxiety, and food security. For employment and income loss we used the following proxy variables: (i) loss of income, defined as the percentage of change in the participant’s total household income in the month prior to the application of the survey compared to February 2020 (pre-COVID), and (ii) employment, was operationalized as a binary variable that identified individuals living in households in which a job or source of income was lost between February 2020 and the month prior to the application of the survey.

The two-item Generalized Anxiety Disorder scale (GAD-2) [16] measures the frequency by which the participant felt during the last 2 weeks: (i) nervous, anxious, or on edge; and (ii) not being able to stop or control worrying. Response options were “never”; “several days”; “more than half of days”; and “almost every day”. An additive score was computed (range 0 to 6), and a cut-off point of 3 or more was classified as having anxiety disorder symptoms. Prior studies have documented that the GAD-2 has appropriate psychometric properties when applied through telephone surveys [13].

Food security was measured with the 8-item adult version of the Latin American and Caribbean Food Secuirty Scale (ELCSA, for its acronym in Spanish). The ELCSA asks if, in the last 3 months, due to a lack of money or other resources, the respondent or any other adult in the household occasionally: (i) worried you might run out of food; (ii) were unable to eat healthy, balanced, and nutritious food; (iii) ate only a few kinds of foods; (iv) skipped breakfast, lunch or dinner; (v) ate less than s/he thought should have; (vi) ran out of food; (vii) were hungry but did not eat; and (viii) went without eating for a whole day. All item-responses are dichotomous (i.e., Yes/No). Through a total summative score, four levels of food security were estimated: food security (total score = 0), mild food insecurity (total score = 1–3), moderate food insecurity (total score = 4–6), and severe food insecurity (total score = 7–8). The telephone application of the ELCSA was recently validated [14].

Time for each cross-section of the survey, a key independent variable, was operationalized as a dichotomous variable. In addition, other control variables included in the analyses were: household socioeconomic status (SES), age, gender, household size and households with children. Household SES was measured with the assets-based AMAI index [3], which combines six household indicators: (i) education level of the head of household; (ii) number of complete bathrooms; (iii) number of cars or vans; (iv) having internet connection; (v) number of household members 14 years or older who are working; and (vi) number of bedrooms. With a summative score and standard cut-off points, SES is classified into seven mutually exclusive categories, ranging from “A/B” to “E”, where A/B represents the highest level and E the lowest SES level, leading to a 4-level variable (i.e. A/B, C, D, E - hereinafter referred to as high, medium, medium-low, and low, respectively). Age and household size were kept as continuous variables, and gender and households with children were transformed into a dichotomous variable.

### Analysis

Descriptive statistics were computed for each cross-section and prevalence across time for each outcome was graphically depicted. Subsequently, we estimated probit models for employment loss and anxiety, a multinomial logistic regression for food insecurity, and an OLS regression for the change in households’ income. All models were adjusted for the previously mentioned covariates. Additionally, for the model estimating anxiety symptoms as the dependent variable we adjusted by food insecurity. For a more explicit interpretation in terms of probabilities, results are presented as the average marginal effects, namely, the marginal effects of each variable on the dependent variable, setting the rest of the variables at their average values. All analyses were estimated in STATA 13 (STATA Corp., 2013) using the svy module to account for the sampling design.

### Results

Table 1 summarizes the sample characteristics for each monthly cross-section between April and August 2020. Resulting from the sampling process, sociodemographic characteristics of participants were similar in each cross-section. Fifty-one per cent of the participants were female; mean age was approximately 40 years; mean household size around 4 members; and approximately 10% of the participants lived in low SES households, 48% in medium-low, 36% medium, and 5% high. In terms of the four dependent variables of interest, the prevalence of anxiety symptoms was relatively stable at approximately 32%. Food security decreased in each cross-section (from 40 to 25%), except in August that showed a similar level as in July. Coupled with this process, food insecurity increased over time and severity level. For example, severe food insecurity increased from 10 to 12%. When compared to pre-COVID household conditions in February 2020, the month prior to each cross-section (i.e. if the cross-sectional survey was collected in May it inquired about April compared to February), approximately a third of the respondents established that someone in their household had lost a job. The highest percentage was in April (37%), showing lower levels thereafter. Similarly, compared to pre-COVID, in the month prior to each cross-section, on average participants reported that their household had seen a reduction in income of 28%, showing a relatively constant trend through the months April to August 2020.

The OLS model assessing the change in total household income with respect to pre-COVID-19 time (Table 2) shows that compared to households in the lowest SES level, the medium and high SES categories were associated with positive and significant increases in the percentage change in income’s household. Additionally, females and households with children were associated with relative reduction in household income of 2.3 and 5.6%, respectively. For each additional person in the household, a reduction in household income of approximately 1.6% was estimated. The time variable showed that compared to April, May and June still exhibited statistically significant declines in the percentage of income lost compared to pre-COVID levels (3.6 and 3.2% respectively), the changes in July and August were also negative although smaller and not statistically significant.

**Table 2:** Associations between employment and income indicators with socioeconomic variables during the COVID − 19 pandemic

	Outcome: Job lost by a household member				Outcome: Change in household income			
	Probit				OLS regression			
	AME	SE	CI	*P*-value	AME	SE	CI	*P*-value
Socioeconomic status (ref: E)								
D	−0.015	0.031	(− 0.075, 0.045)	0.622	0.290	2.029	(−3.689, 4.268)	0.887
C	− 0.105**	0.030	(−0.165, − 0.046)	0.001	6.630**	2.026	(2.659, 10.602)	0.001
A/B	−0.157**	0.036	(−0.228, − 0.085)	< 0.001	14.038**	2.456	(9.224, 18.852)	< 0.001
Age	−0.002**	0.001	(−0.003, − 0.002)	< 0.001	0.017	0.035	(−0.052, 0.086)	0.623
Sex (ref: male)								
Female	0.064**	0.014	(0.035, 0.092)	< 0.001	−2.299*	0.992	(−4.244, − 0.353)	0.021
Households with children (ref: no)								
Yes	0.052**	0.017	(0.019, 0.085)	0.002	−5.554**	1.116	(−7.743, −3.366)	< 0.001
Month (ref: April)								
May	−0.084**	0.028	(−0.138, − 0.030)	0.002	−3.559+	1.963	(−7.406, 0.289)	0.070
June	−0.072**	0.023	(−0.116, − 0.027)	0.002	−3.282*	1.602	(−6.423, − 0.141)	0.041
July	−0.072**	0.023	(−0.118, − 0.026)	0.002	−2.194	1.631	(−5.392, 1.004)	0.179
August	−0.046*	0.023	(−0.092, − 0.001)	0.046	−1.028	1.587	(−4.140, 2.084)	0.517
Household size	0.041**	0.004	(0.0330, 0.049)	< 0.001	−1.567**	0.271	(−2.099, −1.036)	< 0.001

*AME* Average marginal effect, *SE* Standard error, *CI* Confidence intervals. ** = significant at 1%; * = significant at 5%; + = significant at 10%

Table 2 also summarizes the estimations of the Probit model on jobs loss in the household compared to pre-COVID conditions. Compared to those in the lowest socioeconomic category, households with higher SES were significantly less likely to be affected, while females and households with children showed a significant increase in the probability of having lost a job, by 6.4 and 5.2 percentage points, respectively. In addition, for each additional individual in the household, the probability of having lost a job increased by 4.1 percentage points. Compared to April, households surveyed in subsequent months showed a significant association in the probability of having someone losing a job or source of income, although the magnitude tended to be smaller over time – May by 8.4 percentage points, June 7.2, July 7.2 and August 4.6.

In the Probit model predicting anxiety symptoms (Table 3), age and gender (i.e. female) were associated with an increase in the probability of reporting anxiety symptoms, by 0.2 and 8.5 percentage points respectively. The household size variable suggested that for each additional individual in the household, the probability of having anxiety symptoms increases by 0.7 percentage points. The month variable was not a significant predictor, suggesting that anxiety might have increased early in the pandemic (i.e. April), with no modifications over time. On the other hand, there was a positive association between severity of food insecurity and increased probability of anxiety symptoms by 12.8, 27.4 and 42 percentage points for moderate, mild, and severe food insecurity respectively.

**Table 3:** Associations between anxiety symptoms with socioeconomic variables during the COVID − 19 pandemic

	Outcome: Anxiety Symptoms			
	Probit			
	AME	SE	CI	***p***-value
Socioeconomic status (ref: E)				
D	0.049+	0.028	(−0.007, 0.104)	0.084
C	0.043	0.028	(− 0.013, 0.098)	0.129
A/B	0.033	0.039	(−0.042, 0.109)	0.387
Age	0.002**	0.000	(0.001, 0.003)	< 0.001
Sex (ref: male)				
Female	0.085**	0.014	(0.057, 0.113)	< 0.001
Households with children (ref: no)				
Yes	0.016	0.016	(− 0.016, 0.048)	0.319
Month (ref: April)				
May	−0.045+	0.026	(−0.095, 0.006)	0.081
June	−0.013	0.021	(− 0.054, 0.029)	0.553
July	−0.027	0.022	(−0.070, 0.016)	0.212
August	−0.024	0.021	(−0.066, 0.018)	0.254
Household size	0.007+	0.004	(− 0.001, 0.014)	0.080
Food insecurity (ref: security)				
Mild insecurity	0.128**	0.016	(0.097, 0.159)	< 0.001
Moderate insecurity	0.274**	0.023	(0.228, 0.319)	< 0.001
Severe insecurity	0.420**	0.025	(0.371, 0.470)	< 0.001

*AME* Average marginal effect, *SE* Standard error, *CI* Confidence intervals

** = significant at 1%; * = significant at 5%; + = significant at 10%

Table 4 summarize the multinomial logistic regression model estimating food insecurity. The estimations suggested that compared to the lowest socioeconomic category, those in higher SES were associated with larger probabilities of being food secure in a dose-response manner (i.e. medium-low SES 7.9 percentage points, medium SES 30.7, and high SES 53.6, respectively). In a similar way, there was a significant association with lower probabilities of moderate and severe food insecurity with the same type of dose-response between higher SES categories exhibiting lower probabilities. On the other hand, women showed a significant reduction in the probability of food security by 6.9 percentage points, and an increased probability of severe food insecurity by 4 percentage points. In terms of household characteristics, those with children, showed a significant association with decreased probability of food security (3.4 percentage points) and an increased probability of moderate food insecurity (3 percentage points). Moreover, each additional person in the household was associated with a reduction in the probability of food security of 2.9 percentage points, and increased probability of all types of food insecurity (mild 1.3 percentage points, moderate 0.7 and severe 0.9). The time variable suggests an association of constant significant decrease in food security compared to April’s prevalence through each month (May 4.6 percentage points, June 10.5, July 13.5 and August 12.8). Coupled with such estimated decreases in food security, a significant association was reported in a sustained increased of mild food insecurity (8.2 percentage points in May, 7.3 in June, 12.2 in July and 10.1 in August). The effects observed for moderate food insecurity are not significant and vary in direction and magnitude, but those for severe food insecurity are not significant but consistently associated with an increased prevalence.

**Table 4:** Associations between food insecurity with socioeconomic variables during the COVID −19 pandemic

	Outcome: Food insecurity															
	m-logit															
	FS				Mild FI				Moderate FI				Severe FI			
	AME	SE	CI	***p***-value	AME	SE	CI	***p***-value	AME	SE	CI	***p***-value	AME	SE	CI	***p***-value
Socioeconomic status (ref: E)																
D	0.079**	0.023	(0.034, 0.125)	0.001	0.061+	0.032	(−0.001, 0.123)	0.053	−0.093**	0.030	(−0.151, − 0.035)	0.002	− 0.048+	0.025	(− 0.097, 0.001)	0.055
C	0.307**	0.024	(0.260, 0.355)	< 0.001	0.04	0.032	(−0.022, 0.102)	0.208	−0.192**	0.029	(−0.249, − 0.135)	< 0.001	− 0.155**	0.024	(− 0.202, − 0.108)	< 0.001
A/B	0.536**	0.034	(0.470, 0.603)	< 0.001	−0.071+	0.039	(−0.148, 0.005)	0.067	−0.267**	0.030	(−0.326, − 0.208)	< 0.001	− 0.198**	0.024	− 0.244, − 0.151)	< 0.001
Age	0.001**	0.000	(0.000, 0.002)	0.002	−0.001+	0.001	(−0.002, 0.000)	0.084	0.000	0.000	(−0.001, 0.001)	0.893	0.000	0.000	(−0.001, 0.000)	0.342
Sex (ref: male)																
Female	−0.069**	0.013	(−0.095, − 0.043)	< 0.001	0.011	0.016	(−0.020, 0.042)	0.477	0.018	0.013	(−0.007, 0.042)	0.157	0.04**	0.010	(0.019, 0.060)	< 0.001
Households with children (ref: no)																
Yes	−0.034*	0.015	(−0.064, − 0.004)	0.026	0 .000	0.018	(−0.036, 0.035)	0.996	0.03*	0.015	(0.001, 0.059)	0.044	0.004	0.012	(−0.020, 0.028)	0.738
Month (ref: April)																
May	−0.046+	0.025	(−0.095, 0.004)	0.070	0.082**	0.029	(0.026, 0.138)	0.004	−0.051*	0.022	(−0.093, − 0.009)	0.018	0.015	0.019	(−0.023, 0.053)	0.441
June	−0.105**	0.021	(−0.146, − 0.064)	< 0.001	0.073**	0.023	(0.027, 0.118)	0.002	0.018	0.019	(−0.018, 0.055)	0.332	0.014	0.015	(−0.015, 0.044)	0.340
July	−0.135**	0.021	(−0.177, − 0.094)	< 0.001	0.122**	0.024	(0.075, 0.168)	< 0.001	−0.004	0.019	(−0.042, 0.033)	0.814	0.018	0.016	(−0.013, 0.049)	0.249
August	−0.128**	0.021	(−0.169, − 0.087)	< 0.001	0.101**	0.023	(0.055, 0.147)	< 0.001	0.014	0.019	(−0.023, 0.051)	0.467	0.013	0.015	(−0.017, 0.043)	0.405
Household size	−0.029**	0.004	(−0.037, − 0.021)	< 0.001	0.013**	0.004	(0.005, 0.021)	0.002	0.007*	0.003	(0.000, 0.013)	0.038	0.009**	0.003	(0.004, 0.014)	< 0.001

*AME* Average marginal effect, *SE* Standard error, *CI* Confidence intervals, *FS* Food secure, *FI* Food insecure

** = significant at 1%; * = significant at 5%; + = significant at 10%

**Fig. 1:**
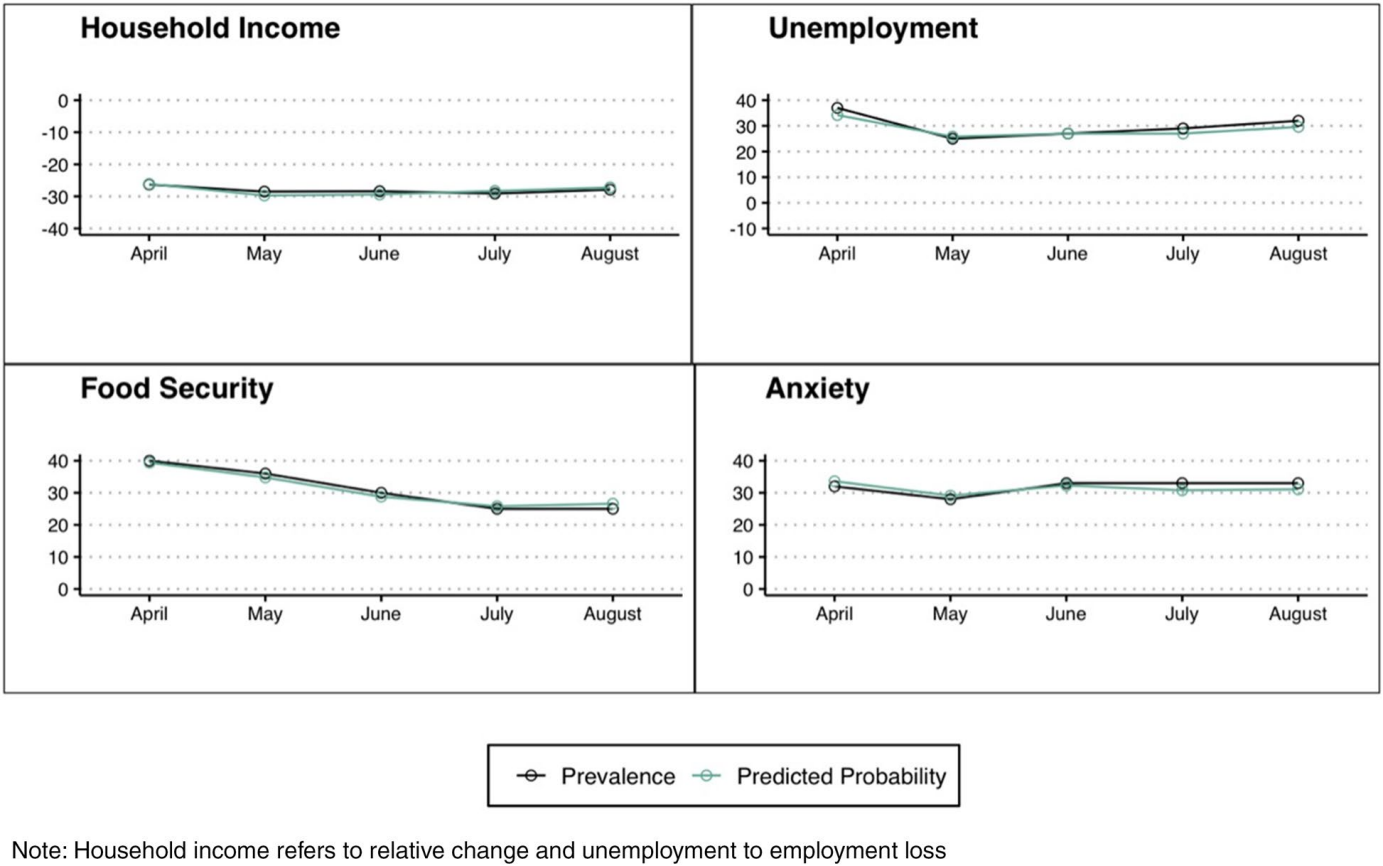
Outcomes and predicted probabilities of well-being measures

Figure 1 summarizes the prevalence as well as the predicted probability of the outcome variables in each of the cross-sections.

As the modelling approach implies several tests within each model and across the models, the null hypotheses could have been rejected by chance. Hence, in Supplementary Table 1 a more conservative approach is presented that accounts for comparisons and multiple tests using STATA qqvalue [27]. This more conservative approach shows similar results to those presented in Tables 2, 3 and 4.

An aspect that emerged from the results of the analyses is if there could be an interplay between different characteristics of the respondent and the well-being outcomes. We ran models with interactions of gender and socioeconomic status (see Supplementary Table 2), but no significant associations were found.

## Discussion

Prior literature has documented the associations between the COVID-19 pandemic and economic and well-being indicators [5, 41]. The current study contributes to such body of evidence by using nationally representative monthly cross-sectional data in Mexico. In terms of economic related indictors, the study showed a sustained effect on job loss at the household level when compared to February 2020 pre-COVID-19 conditions. Even though lockdown measures were relaxed during the summer of 2020, the study showed continued associations with loss of employment. Descriptive data suggested that in April 2020 the average percentage change on household income – comparing March to February 2020 – had already decreased by 26%, and in the adjusted model, all models showed continued negative effects (i.e. larger percental losses), although only statistically significant in May and June. This suggests that income loss persisted and no signs of recovery emerged from the model.

It is fundamental to stress that the Mexican government has not taken actions to safeguard the well-being of households during the COVID-19 pandemic. This is even more worrisome because effects have been larger among households who were already vulnerable prior to the pandemic, such as those with lower SES. Consistent with prior evidence [2, 9], this study highlighted that the impacts on both income and employment were worse among females, as well as in households with children. This is particularly worrisome as it can lead to increasing gender gaps, as well as long-term effects among the affected children [29]. The potential effect on vulnerable households with children could have been minimized through specific subsidies, which despite being costed early in the pandemic to facilitate policy action [39], have not been implemented.

Food security has also been greatly affected by the pandemic in Mexico. Prior analyses have shown that compared to data from national pre-COVID-19 surveys, in April food security had already shown decreased prevalence coupled by an increase in mild food insecurity [14]. This study showed that the effects persisted across the first semester of the pandemic, suggesting a trend of lower levels of food security, which have not returned to pre-COVID-19 levels, and a dynamic process in which initially mild food insecurity increased, but as time has elapsed it has shifted towards increases in more severe levels. Although these trends are not significant in the analysis, if this trend continues, it is likely to become significant in subsequent months. This should be a national policy priority due to the previously documented deleterious associations between more severe levels of food insecurity and health indicators ([31], & [13]). Findings of the study also suggest that such trends can lead to further health and nutrition inequities in households with children and among women.

Food insecurity was associated with higher probabilities of symptoms of anxiety. This is an expected association as food insecurity is a known predictor of mental health conditions [38]. Nevertheless, this relationship is particularly relevant in a situation in which food insecurity is getting worse through time. Unfortunately, in Mexico there was no nationally representative data of pre-COVID-19 anxiety prevalence. The observed prevalence in the ENCOVID-19 cross-sections suggests a high prevalence in April, and was sustained at around the same level throughout the study period (April–August). It can be that increasing food insecurity has been a constant stressor, coupled with lower incomes and lost employment compared to pre-COVID-19 levels. For example, prior studies have found that being a parent in food insecure households facing trade-offs between food and other basic necessities is associated with paternal/maternal stress [15, 21]. The syndemic theory might provide a useful framework in assessing the aggregate effect of several vulnerabilities as it examines mutually enhancing diseases/health determinants under conditions of social inequality [18, 40]. In this sense, the findings might suggest a syndemic process in the deterioration of different well-being indicators during the same period of time. It is of surmount importance to consider such parallel processes to understand the sustained pressure and vulnerability of households, especially among those already facing disadvantages prior to the pandemic. Without an integral social policy, this will magnify economic, health and nutrition related inequities, as well as gender disparities.

Our study had some limitations. Although the ideal design would have been a longitudinal/panel study, in its inception the ENCOVID-19 emerged as an emergency response survey trying to fill the gaps of data that stopped being collected by governmental agencies. This led to initial limited resources. In addition, when designed in March 2020 none of the involved researchers foresaw the extended temporality of the pandemic. Hence, as reality evolved, newer cross-sections were added to keep measuring well-being indicators. Additionally, in the Mexican context these types of surveys were usually collected face-to-face. The pandemic forced the use of a telephone survey that requires shorter versions of scales [6], which in some cases also needed to be adapted and validated [13, 14]. This has led to slight modifications in the measurement of some variables. Lastly, due to the relevance of keeping telephone surveys within a reasonable time-length, and given the pressing need during the early stages of the pandemic to document diverse social and policy issues, some topics – like depression – were not included in every cross-section. Inclusion of variables was based on evidence. For example, in the specific case of mental health the polychoric correlation between depression and anxiety scales was 0.67; among all respondents with symptoms of depression 69% also had symptoms of anxiety, while among those without symptoms of depression 79% did not have of anxiety either. While anxiety is a good proxy of mental health in the current study, it is not the only mental health condition to be documented.

## Conclusions

This study documents the sustained and continued tension in four well-being indicators during the initial 5 months of the pandemic in Mexico, and the increased deleterious association of such process with pre-COVID-19 vulnerabilities according to SES, gender and among households with children. The indicators studied have the potential of syndemic associations that would require multisectoral policy interventions, which are far from the public agenda in the country.

## Supplementary Information

**Additional file 1: Supplemental Table 1.** Comparing *p*-values and q-values of models estimating the associations between wellbeing measures and different socioeconomic indicators during COVID-1.

**Additional file 2: Supplemental Table 2.** Average marginal effects of sociodemographic variables on well-being indicators during COVID (with interaction effects between gender and socioeconomic stats).

## Data Availability

The ENCOVID-19 datasets used during the current study are available in the Zenodo repository, on the following link: 10.5281/zenodo.4602374. The specific STATA Code is available upon request.

## Abbreviations

AMAI: Mexican Association of Market and Opinion Intelligence Agencies (for its acronym in Spanish)
CATI: Computer Assisted Telephone Interviewing software
COVID-19: Severe acute respiratory syndrome coronavirus 2
ELCSA: Latin American and Caribbean Food Security Scale
ENCOVID-19: Survey on COVID-19 Effects on Wellness in Mexican Households (for its acronym in Spanish)
GAD-2: Generalized Anxiety Disorder Scale
INEGI: Mexican National Bureau of Statistics
SDG: Sustainable Development Goals
SES: Socioeconomic status
