# Supplementary material for: Associations of the COVID-19 pandemic with social well-being indicators in Mexico
Source: Int J Equity Health. 2022 May 21;21:74. doi: 10.1186/s12939-022-01658-9 (PMC9123783; doi:10.1186/s12939-022-01658-9)
Supplement: Supplementary file 1 — Additional file 1: Supplemental Table 1. Comparing p-values and q-values of models estimating the associations between wellbeing measures and different socioeconomic indicators during COVID-1. [file 12939_2022_1658_MOESM1_ESM.docx]

Supplemental Table 1. Comparing p-values and q-values of models estimating the associations between wellbeing measures and different socioeconomic indicators during COVID-1

|  | **Job loss** | | **Change in income** | | **Anxiety** | | **Food security** | | **Mild food insecurity** | | **Moderate food insecurity** | | **Severe food insecurity** | |
| --- | --- | --- | --- | --- | --- | --- | --- | --- | --- | --- | --- | --- | --- | --- |
|  | **p-value** | **q-values** | **p-value** | **q-values** | **p-value** | **q-value** | **p-value** | **q-values** | **p-values** | **q-value** | **p-values** | **q-values** | **p-value** | **q-values** |
| Socioeconomic status (ref: E) |  |  |  |  |  |  |  |  |  |  |  |  |  |  |
| D | 0.6221 | 1.0000 | 0.8865 | 1.0000 | 0.0526 | 0.0552 | 0.0552 | 0.6077 | 0.6077 | 0.0018 | 0.0018 | 0.0194 | 0.0552 | 0.6077 |
| C | 0.0005 | 0.0056 | 0.0011 | 0.0118 | 0.2076 | 0.0000 | 0.0000 | 0.0000 | 0.0000 | 0.0000 | 0.0000 | 0.0000 | 0.0000 | 0.0000 |
| A/B | 0.0000 | 0.0002 | 0.0000 | 0.0000 | 0.0667 | 0.0000 | 0.0000 | 0.0000 | 0.0000 | 0.0000 | 0.0000 | 0.0000 | 0.0000 | 0.0000 |
| Age | 0.0000 | 0.0000 | 0.6230 | 1.0000 | 0.0843 | 0.3423 | 0.3423 | 1.0000 | 1.0000 | 0.8930 | 0.8930 | 1.0000 | 0.3423 | 1.0000 |
| Sex (ref: male) |  |  |  |  |  |  |  |  |  |  |  |  |  |  |
| Female | 0.0000 | 0.0001 | 0.0206 | 0.2263 | 0.4771 | 0.0001 | 0.0001 | 0.0016 | 0.0016 | 0.1575 | 0.1575 | 1.0000 | 0.0001 | 0.0016 |
| Households with children (ref: no) |  |  |  |  |  |  |  |  |  |  |  |  |  |  |
| Yes | 0.0018 | 0.0201 | 0.0000 | 0.0000 | 0.9960 | 0.7379 | 0.7379 | 1.0000 | 1.0000 | 0.0437 | 0.0437 | 0.4803 | 0.7379 | 1.0000 |
| Month (ref: April) |  |  |  |  |  |  |  |  |  |  |  |  |  |  |
| May | 0.0022 | 0.0246 | 0.0699 | 0.7684 | 0.0042 | 0.4406 | 0.4406 | 1.0000 | 1.0000 | 0.0183 | 0.0183 | 0.2013 | 0.4406 | 1.0000 |
| June | 0.0015 | 0.0170 | 0.0406 | 0.4463 | 0.0017 | 0.3399 | 0.3399 | 1.0000 | 1.0000 | 0.3323 | 0.3323 | 1.0000 | 0.3399 | 1.0000 |
| July | 0.0020 | 0.0217 | 0.1787 | 1.0000 | 0.0000 | 0.2495 | 0.2495 | 1.0000 | 1.0000 | 0.8142 | 0.8142 | 1.0000 | 0.2495 | 1.0000 |
| August | 0.0464 | 0.5101 | 0.5172 | 1.0000 | 0.0000 | 0.4053 | 0.4053 | 1.0000 | 1.0000 | 0.4671 | 0.4671 | 1.0000 | 0.4053 | 1.0000 |
| Household size | 0.0000 | 0.0000 | 0.0000 | 0.0000 | 0.0024 | 0.0004 | 0.0004 | 0.0042 | 0.0042 | 0.0384 | 0.0384 | 0.4219 | 0.0004 | 0.0042 |
| Food insecurity (ref: security) |  |  |  |  |  |  |  |  |  |  |  |  |  |  |
| Mild insecurity |  |  |  |  | 0.0000 | 0.0000 |  |  |  |  |  |  |  |  |
| Moderate insecurity |  |  |  |  | 0.0000 | 0.0000 |  |  |  |  |  |  |  |  |
| Severe insecurity |  |  |  |  | 0.0000 | 0.0000 |  |  |  |  |  |  |  |  |

|  | 1% significance |
| --- | --- |
|  | 5% significance |
|  | 10% significance |
