# Supplementary material for: Associations of the COVID-19 pandemic with social well-being indicators in Mexico
Source: Int J Equity Health. 2022 May 21;21:74. doi: 10.1186/s12939-022-01658-9 (PMC9123783; doi:10.1186/s12939-022-01658-9)
Supplement: Supplementary file 2 — Additional file 2: Supplemental Table 2. Average marginal effects of sociodemographic variables on well-being indicators during COVID (with interaction effects between gender and socioeconomic stats). [file 12939_2022_1658_MOESM2_ESM.docx]

Supplemental Table 2. Average marginal effects of sociodemographic variables on well-being indicators during COVID (with interaction effects between gender and socioeconomic stats)

|  | **Symptoms of Anxeity** | **Food insecurity** | | | | **Job lost by a household member** | **Change in household income** |
| --- | --- | --- | --- | --- | --- | --- | --- |
|  | **Probit** | **m-logit** | | | | **Probit** | **OLS Regression** |
|  |  | ***FS*** | ***MiFI*** | ***MoFI*** | ***SeFi*** |  |  |
| Socioeconomic status (ref: E) |  |  |  |  | s |  |  |
| D | 0.049+ | 0.077** | 0.067* | -0.097** | -0.047* | -0.02 | 0.141 |
| C | 0.043 | 0.293** | 0.042 | -0.189** | -0.146** | -0.11** | 6.527** |
| A/B | 0.026 | 0.508** | -0.06 | -0.259** | -0.189** | -0.157** | 14.065** |
| Age | 0.002** | 0.002** | -0.001 | 0 | -0.001 | -0.003** | 0.016 |
| Sex (ref: male) |  |  |  |  |  |  |  |
| Female | 0.092** | -0.05** | 0.014 | 0.009 | 0.027** | 0.064** | -2.308* |
| Households with children (ref: no) | 0.019 |  |  |  |  |  |  |
| Yes |  | -0.029+ | 0.002 | 0.028+ | -0.001 | 0.053** | -5.499** |
| Month (ref: April) | -0.05+ |  |  |  |  |  |  |
| May | -0.014 | -0.052* | 0.08** | -0.048* | 0.021 | -0.084** | -3.546+ |
| June | -0.03 | -0.103** | 0.072** | 0.017 | 0.014 | -0.072** | -3.3* |
| July | -0.027 | -0.136** | 0.12** | -0.004 | 0.02 | -0.072** | -2.196 |
| August | 0.007+ | -0.128** | 0.1** | 0.014 | 0.014 | -0.047* | -1.038 |
| Household size |  | -0.026** | 0.013** | 0.006+ | 0.008** | 0.041** | -1.573** |
| Food insecurity (ref: security) |  |  |  |  |  |  |  |
| Mild insecurity | 0.122** |  |  |  |  |  |  |
| Moderate insecurity | 0.263** |  |  |  |  |  |  |
| Severe insecurity | 0.42** |  |  |  |  |  |  |
| Symptoms of Anxiety |  | -0.16** | -0.044** | 0.079** | 0.125** |  |  |
| NSE # sexo |  |  |  |  |  |  |  |
| D # mujer | 0.025 | 0.031 | -0.024 | 0.057 | -0.063 | 0.048 | 2.178 |
| C # mujer | 0.083 | 0.068 | -0.059 | 0.058 | -0.066 | 0.032 | 3.270 |
| A/B # mujer | 0.000 | 0.019 | -0.020 | 0.073 | -0.073 | 0.057 | 3.594 |

FS: food secure, MiFI: mild food insecure, MoFI: moderate food insecure, SeFI: Severe food insecure.

Estimations based on ENCOVID-19. **=significant at 1%; *=significant at 5%; +=significant at 10%
